# Structural Updates to the Implant and Refill Needle of the Port Delivery Platform

**DOI:** 10.1167/tvst.14.4.8

**Published:** 2025-04-07

**Authors:** Dante J. Pieramici, Peter A. Campochiaro, Margaret Chang, Ian Pearce, Carl D. Regillo, Shamika Gune, Nancy Holekamp, Carlos Quezada-Ruiz, Shrirang V. Ranade, Natasha Singh, Geoffrey Wise, Joshua Horvath

**Affiliations:** 1 California Retina Consultants, California Retina Research Foundation, Retina Consultants of America, Santa Barbara, CA, USA; 2 The Wilmer Eye Institute, Department of Ophthalmology, Johns Hopkins University School of Medicine, Baltimore, MD, USA; 3 Retinal Consultants Medical Group, Sacramento, CA, USA; 4 St. Paul’s Eye Unit, Royal Liverpool University Hospital, Liverpool, UK; 5 Wills Eye Hospital, Thomas Jefferson University, Mid Atlantic Retina, Philadelphia, PA, USA; 6 Genentech, Inc., South San Francisco, CA, USA; 7 F. Hoffmann-La Roche Ltd., Basel, Switzerland; 8 Clínica de Ojos Garza Viejo, San Pedro Garza García, Nuevo León, Mexico

**Keywords:** Port Delivery System (PDS), neovascular age-related macular degeneration (nAMD), diabetic macular edema (DME), ocular implant, vitreoretinal surgery

## Abstract

**Purpose:**

The purpose of this study was to report the impact of component-level changes and manufacturing process improvements on septum durability in the ocular implant of the Port Delivery Platform (PD-P), more commonly known as the Port Delivery System with ranibizumab (PDS) with its current formulation.

**Methods:**

Laboratory tests were conducted to determine (1) the bond strength of the septum to the overmold, (2) the amount of force utilized for the refill needle to puncture the septum, and (3) septum durability over 50 years. To simulate multiple refill-exchanges over long-term clinical use, implants were aged at elevated temperature in saline and the septum was repetitively punctured at random locations using the refill needle every 3.5 days, simulating 6 months of use.

**Results:**

Updates to the septum-overmold interface of the implant and manufacturing process improvements doubled the bond strength between the overmold and septum (1.2 N to 2.4 N). Light lubrication of the refill needle reduced needle insertion force into the septum by >50% (1 N to 0.4 N). Together, these modifications increased long-term septum durability, with no septum dislodgements being observed after over 50 years of simulated use.

**Conclusions:**

Structural updates to the PD-P implant and refill needle have met and exceeded performance specifications, mitigating the risk of future septum dislodgement in the updated product.

**Translational Relevance:**

Structural updates to the PD-P implant and refill needle resulted in the ability to withstand the equivalent of over 50 years of simulated use without septum dislodgement and should improve the longevity of the device in clinical use.

## Introduction

The Port Delivery Platform (PD-P) is an intraocular drug delivery system that includes a refillable ocular implant and ancillary devices.^1^ When this implant is used to deliver a customized formulation of ranibizumab 100 mg/mL, it is better known as the Port Delivery System with ranibizumab (PDS).^1^^,^^2^ Once implanted surgically at the pars plana, the PDS requires refill-exchange procedures twice a year to deliver results equivalent to monthly intravitreal injections of ranibizumab in patients with neovascular age-related macular degeneration (nAMD) or diabetic macular edema (DME).^2^^–^^4^ PDS is the only continuous drug delivery system that has shown positive phase III data across three different indications (nAMD, DME, and diabetic retinopathy [DR]),^2^^,^^4^^,^^5^ and was approved for the treatment of patients with nAMD in the United States in 2021 by the US Food and Drug Administration (FDA).^6^ Efficacy experience and surgical learnings over more than 5 years have built confidence in this treatment given the long-term vision and anatomic outcomes seen with continuous delivery of ranibizumab.^7^ Additionally, in clinical trials, the PDS has been demonstrated to be preferred by patients over intravitreal injections of anti-vascular endothelial growth factor (anti-VEGF) monotherapy across three different indications (DR, DME, and nAMD).^8^^,^^9^

**Figure 1.**
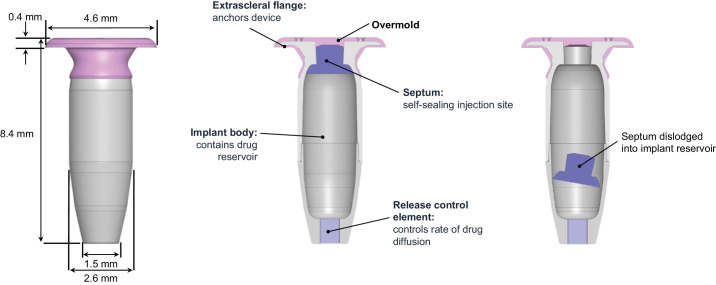
Components of the PD-P implant. Images not to scale. Figure depicts the recalled implant. Images used with permission. Copyright 2023 F. Hoffmann-La Roche Ltd. All rights reserved. PD-P, Port Delivery Platform.

In 2022, incidences of “septum dislodgement” were reported in PDS clinical trials (see the Table; see Supplementary Table S1 for detailed timeline of events).^10^ The septum is a component of the implant that ensures self-sealing of the drug reservoir (Fig. 1, middle panel). In a septum dislodgement incident, the septum is dislodged into the body of the implant (see Fig. 1, right panel).^10^ Once identified, an additional investigation was initiated, which found that implants did not meet the prespecified performance standard of no device failures or malfunctions in ≥97.5% of implants over at least 10 simulated years of use (i.e. 21 punctures).^11^ Due to these findings, Roche/Genentech both voluntarily recalled the PDS ocular implant and insertion tool assembly and paused new implantations in ongoing global clinical trials in October 2022.^12^ The FDA classified this voluntary recall as Class III, wherein use of the product is considered unlikely to cause adverse health consequences.^13^^,^^14^

**Table.** Summary of Reported Cases of Septum Dislodgement in PDS Implants at the Time of Recall

	All^*^	Phase II	Phase III	Commercial
Dates of implantation	Sep 2015–Sep 2022	Sep 2015–Apr 2018	Sep 2018–Aug 2022	Dec 2021–Sep 2022
Patients implanted, *n*	1429	195	1082	189
Total number of refill-exchanges	5198	1726	3455	17
Maximum refill-exchanges in a patient	20	20	8	1
Number of septum dislodgements	33	0	33	0
Septum dislodgement rate per 100 implants	2.3%	0%	3.0%	0%

PDS, Port Delivery System with ranibizumab.

Data as of August 31, 2022.

* Includes patients implanted with phase II, phase III, and commercial implants.

Along with the PDS ocular implant, the voluntary recall also included the insertion tool assembly, the ranibizumab 100 mg/mL drug vial, and the initial fill needle (lot numbers 3499188 and 3523071), which were sold together.^13^ The voluntary recall did not affect either the refill needle or the vial of ranibizumab 100 mg/mL for the ocular implant refill, and eligible patients who already had an implant but had not experienced septum dislodgement continued to receive refill-exchanges.^12^ By October 31, 2023, there were 1466 patients who were implanted with the PDS and had received a total of 7504 refill-exchanges (with no individual patient receiving more than 21 refill-exchanges; data on file). A total of 78 (5.3%) cases of septum dislodgements had been reported, with all instances involving implants from the phase III lots.^15^ No septum dislodgements have been reported as of August 2024 in either the phase II or commercial implants (data on file).

To further investigate the causes for septum dislodgement, additional laboratory testing of the implants was initiated. Septum durability was measured in a laboratory simulation of long-term clinical use following the American Society for Testing Materials (ASTM) Standard Guide for Accelerated Aging of Sterile Barrier Systems and Medical Devices (ASTM F1980 – 21)^16^ and using a Q10 factor of 2.5 for similar silicone material per Ou et al.,^17^ and is described herein (see the Methods section). In this simulation, the implants also did not perform to the preset quality standards, with less than 97.5% of septa remaining intact after 10 simulated years of implantation.^11^

**Figure 2.**
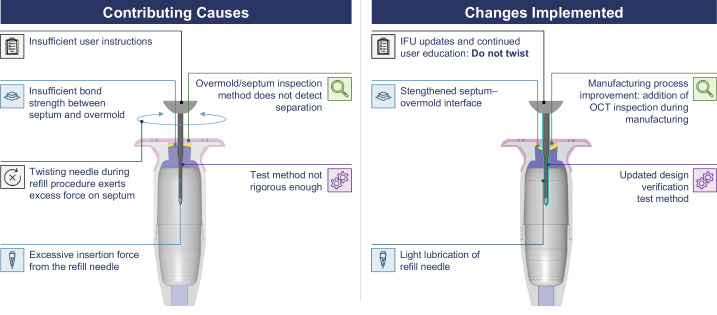
Root cause analysis. Images not to scale. Images used with permission. Copyright 2023 F. Hoffmann-La Roche Ltd. All rights reserved. IFU, instructions for use; OCT, optical coherence tomography.

**Figure 3.**
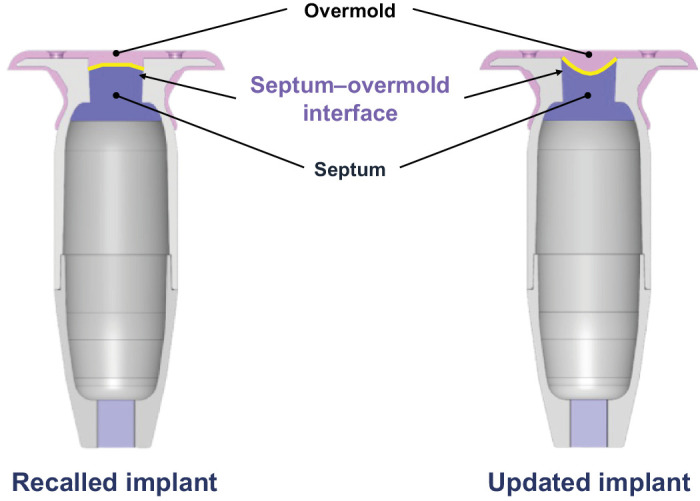
Component-level changes to the PD-P implant. Images not to scale. Images used with permission. Copyright 2023 F. Hoffmann-La Roche Ltd. All rights reserved. PD-P, Port Delivery Platform.

Subsequently, a root cause analysis to pinpoint the cause of the failure was launched. In the analysis of these experiments, the leading factors contributing to septum dislodgement were identified to be (1) insufficient bonding between the septum and the overmold and (2) excessive septum deformation caused by the refill needle insertion (Fig. 2).^15^ Although twisting during the refill-exchange procedure was not found to be a root cause of septum dislodgement, twisting can cause trauma to the conjunctiva and Tenon's capsule, predisposing conjunctival adverse events; therefore, twisting of the refill needle is highly discouraged.^18^ To address the identified issues, component-level changes and manufacturing process improvements were made. Specifically, the interface of the septum and the overmold was changed to increase the contact area (Fig. 3). Additionally, the amount of epoxy used to bond the septum to the implant body was reduced to avoid excess epoxy exposure to the septum and a second oven treatment cycle was implemented to vaporize any uncured epoxy components. To reduce the force needed to insert the refill needle into the overmold and septum, the updated refill needle was lightly coated with a very small amount of silicone-based lubricant that was immobilized on the refill needle's exposed cannula surfaces through a partial crosslinking process. Detection and quantification with nuclear magnetic resonance determined that the amount of silicone found in the combined volume of ranibizumab administered in approximately 50 refill-exchanges made with the updated refill needle was negligible.

After these component updates, performance testing was conducted to measure the septum-overmold bond strength, the force required to insert the refill needle, and the durability of the septum in the ocular implant. In this paper, we describe the test methodology, laboratory simulation model, and results of the performance testing of the updated PD-P implant and updated refill needle.

## Methods

### Study Design

In the laboratory, the bond strength of the septum to the overmold was tested in both the voluntarily recalled commercial implant (hereafter referred to as the “recalled implant”) and the updated implant. In separate laboratory tests, the force of the needle puncturing the septum was measured for both the original refill needle (hereafter referred to as the “prior refill needle”) and the updated refill needle. Last, septum durability was measured in a laboratory simulation of long-term clinical use.

### Testing

#### Septum to Overmold Bond Strength Test

The bond strength of the septum to the overmold was measured with the overmold separation test, which utilized a tensile testing system equipped with a 50 N Load Cell. After separating the overmold from the sides of the implant body (such that the overmold material stands above the flange by itself and is bonded only to the septum), the two overmold folds on either side of the septum-overmold bond were secured in the test fixture overmold clamp (see Supplementary Fig. S1, Supplementary Video S1). The remaining part of the implant was secured in a lower fixture. Using a tensile test method, the overmold was displaced up to 10.00 mm at 80.00 mm/min, and the maximum force exerted was measured with 0.1 N precision at 5.00 ms intervals. Bond strength was measured in 30 recalled implants and 30 updated implants.

#### Refill Needle Insertion Force Test

The insertion force required for the refill needle to puncture the septum was measured with a tensile tester equipped with a 10 N Load Cell. To measure peak required force, the implant was secured in the test fixture clamp, the refill needle was attached to a Luer Mount, and the needle was aligned approximately 1 mm above the implant's septum (see Supplementary Fig. S2, Supplementary Video S2). Using a compression testing mode, the refill needle was displaced 4.30 mm at 80 mm/min into the implant septum, and the maximum force exerted was measured to 0.2 N precision at 50 ms intervals. Insertion force into the recalled implant was measured in 26 prior refill needles and 26 updated refill needles.

#### Simulation Model of Long-Term Clinical Use and Septum Durability Test

As mentioned previously, to measure septum durability in a laboratory simulation of long-term clinical use, ASTM F1980 – 21^16^ was followed, using a Q10 factor of 2.5 for similar silicone material per Ou et al.^17^ Implants were aged for ∼3.5 days at 80°C in phosphate-buffered saline (where the saline storage simulates vitreous exposure). The aged septum was repetitively punctured using the refill needle every 3.5 days (simulating multiple refill-exchange procedures). Thus, in this model system, 1 week of aging with two punctures simulated 1 year of clinical use.

Because the force exerted on the septum increases with increasing speed of needle insertion, to assess long-term septum durability under the least-favorable conditions, the refill needle was inserted at 10 mm/s – a speed slightly faster than used by clinicians in simulated refill procedures (see Supplementary Fig. S3, Supplementary Video S3). The refill needle was inserted at random locations across the aged septum, including up the very edge of the septum where the needle insertion places the greatest stress on the septum. Although the nominal insertion angle of the refill needle is perpendicular to the septum, the needle is never perfectly straight in practice. Extensive testing has shown that inserting the refill needle at up to 20 degrees deviation from perpendicular does not change the force required to insert the needle into the septum.

At test start, one puncture was made with an initial fill needle to simulate the initial fill procedure followed in clinical use; all subsequent punctures were made with refill needles. Every puncture was exerted at near to the maximum force expected during normal clinical use. After each puncture, the aged implant was tested for leakage under air pressure and checked for septum dislodgement. In this way, the septum durability of the recalled implant was tested with both the prior refill needle (*n* = 188) and the updated refill needle (*n* = 188), whereas the updated implant was tested only with the updated refill needle (*n* = 119).

### Outcomes and Statistics

The primary outcomes measured were bond strength of the septum to the overmold, the maximum force required to insert the needle into the septum, and the durability of the septum in a laboratory simulation of long-term clinical use. Outcomes are reported with descriptive statistics.

## Results

### Bond Strength

**Figure 4.**
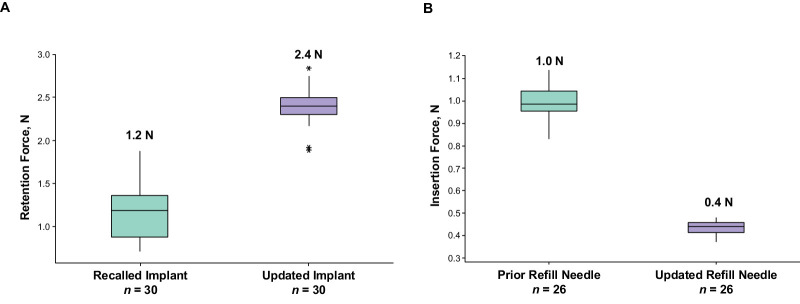
Updates to PD-P components. (**A**) Bond strength between the septum and overmold. The retention force between the septum and overmold is doubled. (**B**) Insertion force of refill needle into recalled implant. Updated refill needle reduced insertion force by more than 50%. PD-P, Port Delivery Platform. *An outlier more than 1.5 times the interquartile range below the first quartile or more than 1.5 times the interquartile range above the third quartile.

Implant manufacturing updates doubled the bond strength between the overmold and septum (1.2 N with recalled implant [*n* = 30]; to 2.4 N with updated implant [*n* = 30]; Fig. 4A).

### Insertion Force

Light lubrication of the refill needle reduced the force required to insert the needle into the septum by more than half (1 N with prior refill needle [*n* = 26]; to 0.4 N with updated refill needle [*n* = 26]; Fig. 4B).

### Septum Durability

**Figure 5.**
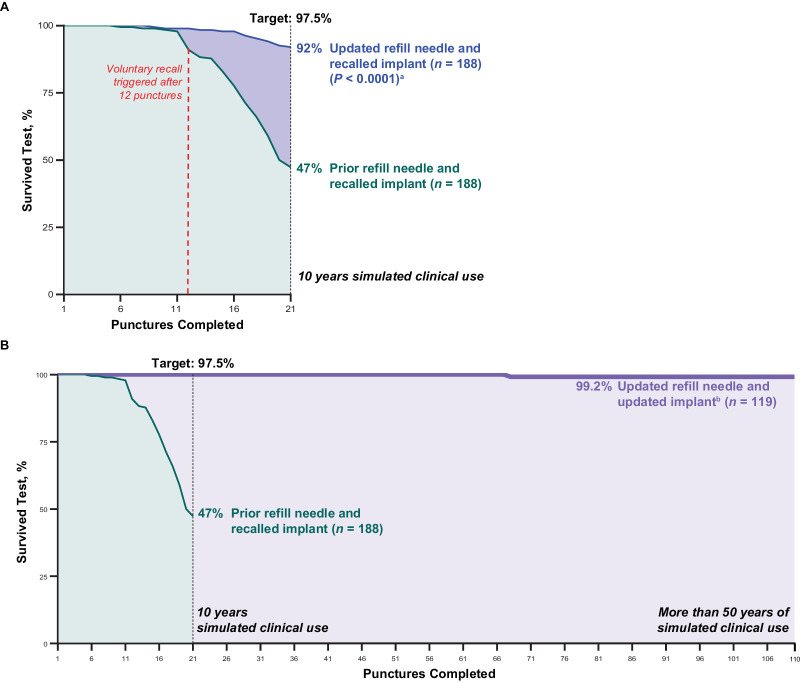
Septum durability testing. (**A**) Recalled implants with prior refill needle (*green curve*) and updated refill needle (*blue curve*). (**B**) Updated refill needle tested with updated implant (*purple curve*). Laboratory tests were conducted to determine long-term septum performance in which implants were aged at elevated temperature in saline and the septum was punctured with the refill-exchange needle every 3.5 days. ^a^At 21 punctures, significantly more septa in the recalled implants survived when punctured with the updated refill needle versus the prior refill needle (Pearson chi square). ^b^One sample failed after puncture 68 by leaking when pressurized through the hole created in the septum by the refill needle; the septum did not dislodge.

In the laboratory septum durability testing in the recalled implant with the prior refill needle, 9.0% of implants had failed by 12 punctures, triggering the voluntary recall of these implants mentioned earlier. At the prespecified end point of 21 punctures, simulating 10 years of clinical use, only 47% of the recalled implants had survived (Fig. 5A). When the recalled implant was punctured with the updated refill needle, 92% of the recalled implants survived after 10 years of simulated use (see Fig. 5A).

When the updated implant was punctured with the updated refill needle, 100% of the updated implants remained intact after 10 years of simulated use (Fig. 5B). At study completion with 110 punctures with the updated needle (simulating over 50 years of clinical use), no septum dislodgements were seen in the updated implants. After 68 punctures with the updated needle, one updated implant failed by leaking when pressurized (although the septum remained intact).

## Discussion

Laboratory testing has established that the implemented component-level changes and manufacturing process improvements have doubled septum-overmold bond strength, whereas light lubrication of the refill needle reduced the insertion force by more than 50%. Septum durability testing demonstrated that the updated PD-P implant exceeds prespecified Roche/Genentech specifications of 97.5% of septa remaining intact after 21 punctures (10 years), with no septum dislodgements being detected over 110 punctures (over 50 years of simulated use). Furthermore, as of July 24, 2024, no cases of septum dislodgement have been reported in the phase II-implanted patients (*n* = 195) who have received up to 20 refill-exchanges over an average of 6.2 years of follow-up, nor have any been reported in commercial-implanted patients. There were manufacturing process changes between phase II and phase III (including more automation to ramp up supply), which may have contributed to the weaker bond between the septum and overmold observed in phase III lots.

In the septum durability test, aging the implants at 80°C for a minimum of 2.94 days would simulate 6 months at body temperature. In this investigation, the implants were aged for 3.4 days on average. As a result of this additional aging, the recalled implants failed on average two or three punctures earlier than they had in clinical trial use, confirming that the laboratory simulation is slightly more aggressive than actual clinical use. This suggests that septum dislodgement in the updated implant will be very rare.

Although there have been some concerns regarding the accumulation of silicone microdroplets with the use of silicone-lubricated syringes for intravitreal injections,^19^^,^^20^ the very small amount of silicone used on the updated refill needle was immobilized and only a miniscule amount accumulated after more than 50 simulated refill-exchanges in a laboratory environment. Furthermore, unlike with intravitreal injections, the refill needle does not enter the vitreous and is instead inserted directly into the implant via the septum. This suggests that silicone microdroplets are highly unlikely to accumulate within the vitreous throughout the duration of treatment with the implant.

Modification of ophthalmic devices after approval are relatively common, with one review showing that 86% (144 out of 168) of original ophthalmic devices approved by the FDA between 1979 and 2015 were modified more than once after approval, with manufacturing alterations being the most frequent type of revision.^21^ Devices as ubiquitous as glaucoma drainage implants have been optimized incrementally over time, as have the surgical techniques used to place them.^22^^,^^23^ The modifications described here follow a similar approach, optimizing the refill needle and implant to increase septum durability. Similarly, the associated surgical techniques continue to evolve and learnings continue to inform surgical implantation education and technique to improve patient outcomes.^24^

Although septum dislodgement did disrupt the ability to continue treatment with the device, it is worth noting that no safety issues were reported due to this event. In clinical trials treating nAMD, DR, and DME, the safety profile of PDS has been manageable and well-understood.^4^^,^^5^^,^^7^ Patient preference for this treatment versus intravitreal injections remains high, with the majority of patients electing to have the malfunctioning device removed and replaced with the updated implant.

Technical updates to the PD-P implant and refill needle have successfully mitigated the risk of septum dislodgement, with the updated implant and updated refill needle exceeding performance requirements. The updated PDS implant and updated refill needle received FDA approval in July 2024,^25^ and since then have been reintroduced in the United States for patients with nAMD. Before this, implantations in clinical studies further investigating the PDS resumed worldwide in February 2024.^26^^–^^28^ A clinical observation study of eight physicians performing 34 uses of the updated refill needle found they were able to use the updated lightly lubricated needle to successfully perform a refill-exchange in accordance with the instructions for use with no unexpected adverse events, supporting the reintroduction of the PDS to clinical practice in the United States. Supplementary videos show one retina specialist performing the refill-exchange procedure on the same patient using the prior refill needle and the update refill needle (see Supplementary Video S4).

Implantations in trials investigating the PD-P have also resumed, including in the Burgundy trial (NCT04567303),^29^ which is a phase I/II study investigating a different molecule, zifibancimig, a DutaFab targeting both VEGF and angiopoietin-2 specifically designed for continuous delivery via the ocular implant to treat retinal diseases in patients with nAMD. The PD-P was designed to address the unmet need for treatment strategies in nAMD and diabetic eye diseases by reducing the burden associated with frequent intravitreal anti-VEGF therapy while maintaining clinical benefits. By combining these component and manufacturing updates with the purposeful evolution of the surgical techniques involved in implantation and the refill-exchange procedure based upon continually growing surgical experience and meticulous analyses of these techniques, the PD-P may support better patient outcomes in clinical practice compared with those achieved with currently available anti-VEGF therapies, as it can provide the same benefits as monthly injections with a reduced treatment burden of only two refill-exchanges per year in patients with nAMD or DME. As the first treatment platform designed for continuous delivery, the PD-P has the potential to shift the treatment paradigm for chronic retinal diseases.

## Supplementary Material

Supplement 1

Supplement 2

Supplement 3

Supplement 4

Supplement 5

Supplement 6

Supplement 7

Supplement 8
