# Supplementary figures and images for: Structural Updates to the Implant and Refill Needle of the Port Delivery Platform
Source: Transl Vis Sci Technol. 2025 Apr 7;14(4):8. doi: 10.1167/tvst.14.4.8 (PMC11980950; doi:10.1167/tvst.14.4.8)

**Supplemental Figure 1.** Grip of Overmold During the Septum Bond Test

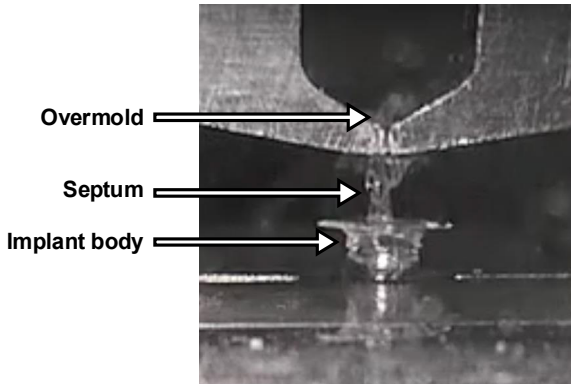

Supplement: Supplement 1 [file tvst-14-4-8_s001.pdf]

**Supplemental Figure 3.** Apparatus for Septum Durability Test

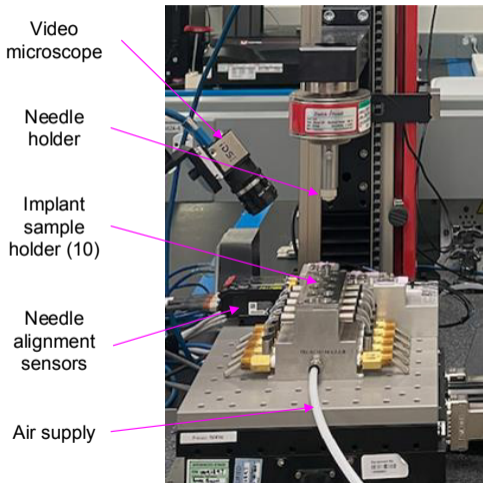

Supplement: Supplement 3 [file tvst-14-4-8_s003.pdf]
