# Supplementary material for: Structural Updates to the Implant and Refill Needle of the Port Delivery Platform
Source: Transl Vis Sci Technol. 2025 Apr 7;14(4):8. doi: 10.1167/tvst.14.4.8 (PMC11980950; doi:10.1167/tvst.14.4.8)

Supplemental Figure 2. Alignment of the Refill Needle Above the Implant Septum for Insertion Force Test

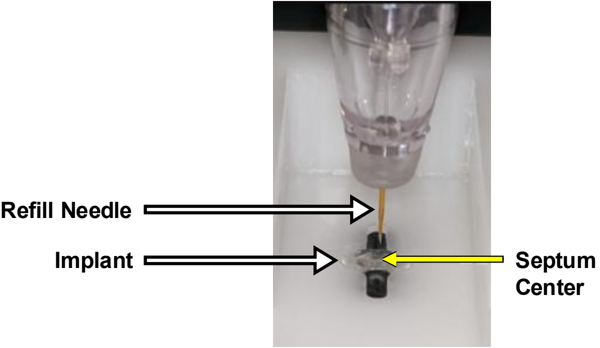

Supplement: Supplement 2 [file tvst-14-4-8_s002.pdf]
