# Supplementary material for: Structural Updates to the Implant and Refill Needle of the Port Delivery Platform
Source: Transl Vis Sci Technol. 2025 Apr 7;14(4):8. doi: 10.1167/tvst.14.4.8 (PMC11980950; doi:10.1167/tvst.14.4.8)
Supplement: Supplement 4 [file tvst-14-4-8_s004.pdf]

Supplementary Tables

Supplementary Table 1. Timeline of Events

July 2021	**First Report** of septum dislodgement
February 2022	Detailed technical investigation (root cause analysis) initiated
	**Safety Memo** issued to inform all investigators about the observations of septum dislodgement<br>**14 cases** out of **~1195 implants** and **~4009 refill-exchanges** across all PDS studies
March 2022	Septum dislodgement data reported at retina congresses
April 2022	**US Prescribing Information** updated to include information about septum dislodgement
May 31, 2022	**Dear Investigator Letter** issued to inform investigators about the decision to replace unused phase 3 implants with those from the commercial lots and provide guidance on patient monitoring, management, and case reporting
October 2022	**Dear Investigator Letter** issued to advise that new PDS implantations had been paused as the PDS implant did not meet the filed specifications for intended use
	**Dear Health Care Provider Letter** issued by Roche/Genentech to inform US HCPs about the **company's decision to initiate a voluntary recall of the PDS ocular implant and the initial fill kit**
	Initiated prototyping activities related to updating the PD-P implant and refill needle
November 2023	Changes at the component and manufacturing level verified with new additional quality controls were implemented to ensure implants meet prespecified performance specifications, mitigating the risk of septum dislodgement
	Septum remained intact after 21 punctures (10 years of simulated clinical use)
	**Dear Investigator Letter** issued to announce the resumption of PDS implantations across all ongoing clinical trials
January 2024	First use of updated (lightly lubricated) refill needle (replacing prior refill needle) in the Clinical Observation Study

February 2024	First implantation with updated implant (utilizing the explant and reimplantation procedure) in clinical trial setting
March 2024	First patient implanted in Burgundy trial (NCT04567303) with updated implant
April 2024	First implantation with updated implant in Velodrome trial (NCT04657289)
	Septum durability testing reached 66 punctures with no septum dislodgements
July 2024	Reintroduction in the United States for patients with nAMD in the commercial setting
July 22, 2024	First implantation in a new patient with updated implant in commercial setting
September 2024	Septum durability testing reached 110 punctures with no septum dislodgements

nAMD, neovascular age-related macular degeneration; PDS, Port Delivery System with ranibizumab.
